# Blinding in trials of interventional procedures is possible and worthwhile

**DOI:** 10.12688/f1000research.12528.2

**Published:** 2018-01-30

**Authors:** Karolina Wartolowska, David Beard, Andrew Carr

**Affiliations:** 1 Nuffield Department of Primary Care Health Sciences (NDPCHS), Radcliffe Observatory Quarter, Woodstock Road, Oxford, OX2 6GG, UK; 2 Botnar Research Centre, Nuffield Department of Orthopaedics Rheumatology and Musculoskeletal Sciences (NDORMS), Windmill Road, Oxford, OX3 7LD, UK

**Keywords:** blinding, RCT, randomised controlled trials, placebo, surgery, procedures, non-pharmacological

## Abstract

In this paper, we use evidence from our earlier review of surgical randomised controlled trials with a placebo arm to show that blinding in trials of interventional procedures is feasible. We give examples of ingenious strategies that have been used to simulate the active procedure and to make the placebo control indistinguishable from the active treatment. We discuss why it is important to blind of patients, assessors, and caregivers and what types of bias that may occur in interventional trials. Finally, we describe the benefits of blinding, from the obvious ones such as avoiding bias, as well as less evident benefits such as avoiding patient drop out in the control arm.

## Introduction

The aim of a trial is to produce unbiased evidence. As randomised controlled trials (RCTs) with a placebo arm control for many types of bias and have high internal validity they are regarded as a reliable method of demonstrating treatment efficacy ^1, 2^. RCTs of interventional procedures are rare ^3– 6^, partly because they are challenging ^7^; however, they are not impossible to perform, even if they involve a placebo arm ^8^. In this paper we discuss why trials should be blinded and summarise methods that have been used to achieve blinding in the published placebo-controlled trials of interventional procedures.

Blinding in interventional trials is often necessary because, nowadays, many procedures are performed to reduce pain and improve function, and quality of life. Pain, function and quality of life are sometimes regarded as preferable outcome measures because they reflect patients’ needs and point of view ^9^. However, as these outcomes depend on patients’ subjective perception, they are prone to bias and may lead to an exaggerated treatment effect in open-label trials ^10– 13^. Using subjective outcomes in an open-label study undermines its internal validity because it makes it impossible to determine how much of the reported effect is related to the investigated treatment and how much is related to various forms of bias.

It is important to note that controlling for bias comes at a price. Because of the standardised conditions under which they are performed, the uncertainty of treatment allocation, and the presence of assessors, RCTs differ from everyday patient care; therefore, they often have low external validity ^14^. Moreover, the size of the effect in blinded trials tends to be smaller than in clinical practice because of the inherent uncertainty of the treatment allocation in a trial ^15^.

## Definitions

In this paper, we have defined surgery like in our previous publication ^8^ as “any interventional procedure that changes the anatomy and requires a skin incision or the use of endoscopic techniques; dental studies were excluded. We used the term placebo to refer to a surgical placebo, a sham surgery, or an imitation procedure intended to mimic the active intervention; including the scenario when a scope was inserted and nothing was done but patients were sedated or under general anaesthesia and could not distinguish whether or not they underwent the actual surgery” ^8^. Also the type of outcomes was defined in the same way as in our earlier paper ^16^ i.e., “Outcomes were classified as “subjective”, i.e., patient-reported and depending on the patients’ perception and cooperation, “assessed”, i.e., subjective ratings by external assessors and depending upon their judgment, and “objective”, i.e., measured using devices or laboratory tests and independent of patients’ or observers’ perception, for example, weight.”

## Blinding of patients, surgeons, outcome assessors, and care-givers

### Blinding of patients

Blinding means concealing the treatment allocation from patients and any other people involved in the trial who may bias the results of the trial by knowing which groups the patients were randomised to.

Blinding of patients prevents reporting bias in patient-reported measures. For example, it has been demonstrated that non-blinded patients may exaggerate the effects size by 0.56 standard deviations and that the effect is even larger in studies on interventional procedures, such as acupuncture ^17, 18^. This bias may be caused by patients’ expectations of treatment effect and information given to them before the treatment ^19^. Patients may also report symptoms depending on their “hunches” about treatment being effective or they may give answers they believe are “correct” or expected from them, for example, because it would have been impolite not to report improvement ^17^. Therefore, it has been suggested that patients should be blinded whenever possible ^17^.

Blinding of patients also reduces adherence bias, i.e. patients in the control group not following the protocol. It may also prevent so called “contamination of the control group”, i.e. seeking additional treatment outside the trial and receiving concomitant treatment. Blinding also improves patient retention in the trial. Risk of attrition in blinded trials is about 4% whereas in non-blinded trials it is 7% ^17^. Specifically, in placebo-controlled surgical trials, subject retention is often reported as “excellent” ^20^, and in our analysis the withdrawal rate was low (4%) and comparable between the treatment and the placebo arm ^21^.

### Blinding of surgeons

Unlike drug trials, in which the physician gives a tablet prepared somewhere else, the surgeon has to perform a specific procedure considered to be therapeutic; therefore, blinding of surgeons may not always be possible.

There have been attempts to blind surgeons, for example, a surgeon inserted a catheter under fluoroscopic guidance and handed over the procedure to a technician who delivered the radiofrequency energy (or not) according to the allocation ^22^. In other trials, a palatal implant delivery system was prepared by the manufacturer to either contain the implant or not, which allowed for blinding of surgeons ^23, 24^.

### Blinding of outcome assessors

In 81% of placebo-controlled surgical trials both patients and assessors were blinded ^21^. It has been demonstrated that, non-blinded assessors of subjective outcomes cause less bias in trials than non-blinded patients reporting their symptoms ^25^. Blinding of assessors prevents observer-related bias, detection bias, and the Pygmalion effect. The Pygmalion effect, also called the Rosenthal effect, refers to a situation when investigators looking for a particular response are predisposed to interpret the result in a way that shows the response they expect, even if it is objectively absent ^26^. Interestingly, a study by Hróbjartsson and colleagues demonstrated that non-blinded assessors were over-optimistic and “over-rated” patients in the treatment group rather than “under-rated” patients in the control group ^27^. To minimise the assessor-related bias, in some trials the assessment was done by people not involved in the surgery, for example, by staff at another hospital ^28^, or by a pathologist blinded to the treatment allocation ^29^.

### Blinding of care-givers

Apart from blinding patients and assessors, it is important that care-givers and clinical or research staff also do not know patient treatment allocation, because their behaviour and attitudes may influence patient responses ^30– 32^. Patient-clinician interaction plays an important role in treatment response, and patients in trials do better as they get more attention and time from clinical staff than patients receiving standard care ^33, 34^. Therefore, the interactions between patients and the trial team should be standardised so that the “treatment context” (similar attention from doctors, expectations, and settings) is comparable between the groups.

## Strategies used to maintain blinding in interventional placebo-controlled trials

A placebo-controlled RCT is a special type of a trial, in which one of the control arms involves an imitation procedure that seems identical but does not involve the crucial element believed to be "the cure". A placebo arm is necessary to demonstrate that the observed improvement is really caused by the investigated procedure as it controls for the effects of receiving treatment other than the crucial surgical element.

It is often difficult to determine what is a specific and what is a non-specific effect in a trial ^34, 35^, and to disentangle a placebo response from response bias or the effect of patient-doctor interactions ^36^. It is beyond the scope of this review to discuss definitions of placebo ^1, 36^. Whether something is or is not a placebo depends on the intervention and chosen outcome variables ^1^, but in order for blinding to be successful, the control procedure has to be as similar as possible to the investigated procedure ^34^. Interventional trials differ from drug trials as they require access to the anatomical structure of interest; therefore, they involve a skin incision or an insertion of a scope.

In many published trials, blinding during the surgery was straightforward because patients were under general anaesthesia or heavy sedation and, therefore; unaware of the details of surgical procedures. In such trials, only the surgical wound had to be similar in both groups. Some studies did not add any placebo procedure but simply omitted part of interventional procedure, for example, in the trial by Stone and colleagues, all patients underwent a percutaneous coronary intervention and maximal medical therapy but only patients in the active arm also had percutaneous transmyocardial revascularisation ^37^.

When light sedation or local anaesthesia were used, surgical staff had to simulate the actual intervention to preserve the blinding. The complexity of a surgical procedure made blinding challenging, and ingenious ideas were required to make the real and placebo interventions indistinguishable.

### Imitation of incision/ surgical access point

If a procedure requires open surgery, then it leaves an obvious mark where the incision has been made, which has to be imitated in the placebo group. There have been very few trials involving full-skin incision, in both the surgical and placebo arms. In the seminal trials on internal mammary artery ligation ^38, 39^ a skin incision was made to expose the arteries in all patients, but no ligation was made in patients in the placebo group. Similarly, Guyuron and colleagues used a skin incision to expose superficial nerves and muscles, which were cut during the active surgery, but in the placebo group, the integrity of these structures was maintained ^40^. Trials investigating transplantation of dopaminergic neurones as a treatment for Parkinson’s disease not only required skin incision but also burr holes in the skull, but in the placebo group the burr holes did not penetrate the dura matter ^41, 42^.

Most of the published placebo-controlled surgical trials used minimally-invasive methods to access the structure of interest. For example, the placebo procedure involved laparoscopy but without ablation ^43^, endoscopy without radiofrequency energy delivery ^44^, bronchoscopy without radiofrequency energy delivery ^45^, or bronchoscopy without a valve placement ^46^. Therefore, most of the studies required a small incision to mimic the portals created during the laparoscopy or arthroscopy, or to mimic the incision through which an intravascular catheter was inserted ^47^. Interestingly, Sutton and colleagues used three incisions in both groups, so that patients could not tell apart a diagnostic laparoscopy from a laparoscopic surgery; even though the third instrument port was not necessary in the placebo group ^48^. Trials using endoscopy and bronchoscopy were even easier to blind as natural orifices were used to insert the scope, and the incision or actual procedure site was not visible to patients, care-givers, and assessors.

### Simulation of an interventional procedure

Typically, the preparation for the placebo and the active procedure was as similar as possible and imitated the visual, auditory, and physical cues ^49– 52^. In order to mimic the sounds, surgeons were required to talk through the procedure steps ^53^, ask for instruments ^54, 55^, use suction ^55^ or ask for a laser or other device to be activated, even though it was not used in the placebo group ^56– 60^.

Clinical staff performing the intervention were screened from the patients’ view ^61^, either by a surgical drape ^59^ or by arranging the operating room in a way that the patient could not see the procedure ^51^. In a trial by Stone and colleagues, patients were heavily sedated and wore opaque goggles ^37^. In a trial by Maurer and colleagues, the manufacturer delivered tools that looked identical but those for the placebo group did not contain an implant, which allowed for blinding of patients and clinical staff ^24^.

Surgeons also attempted to imitate sensory cues, for example, by manipulating the knee as if the actual arthroscopy were performed ^55^, injecting saline to imitate tidal irrigation ^20^, or by splashing saline on the knee to simulate lavage ^52^. In a trial on meniscectomy, Sihvonen and colleagues used a mechanised shaver (without the blade) pushing it firmly against the patella to simulate the sensations the patient would experience during the surgery ^55^. In a trial on intragastric balloon for obesity, operators manipulated the endoscope as during the balloon insertion to create the sensation of resistance in the stomach ^60^.

Even smell during the surgery was imitated to make the placebo procedure indistinguishable from surgery. For example, in the trial by Deviere and colleagues there were concerns that patients could have known the allocation because the copolymer used in the active arm had a distinct smell ^62^. In trials on vertebroplasty, a container with cement was opened during placebo procedure to help with blinding by imitating the smell ^49, 63^.

### Inactive nature of the placebo

It is important that the procedure used for blinding does not have any therapeutic effect. For example, the results of the vertebroplasty trials ^43, 63^ were criticised because the elements of placebo procedure could have had an effect on the reported pain, namely, a potential pharmacological anaesthesia due to injection of an anaesthetic into the facet capsule and periosteum ^64^.

On the other hand, the procedure used for blinding may have diagnostic use, as with diagnostic laparoscopy ^43, 48, 65^ or diagnostic laparoscopy with biopsy ^66^. In the trial by Sihvonen and colleagues, all participants underwent diagnostic arthroscopy, but only after they had been confirmed to be eligible for inclusion in the trial was the envelope with the assignment opened and the assignment revealed to the surgeon ^55^.

### Duration of the procedure

Many trials specifically stated that the duration of procedure in the surgical and control arms were matched, either by imitating the elements of the surgical procedure or by keeping all patients in the operation room for the same duration of time ^41, 45, 52, 54, 55, 67, 68^. However, in some trials, the placebo procedure was shortened in comparison to the actual surgery because it was believed it would have been ethically unacceptable to prolong the placebo intervention ^56, 62^.

### Additional procedures that may reveal allocation

Interventional treatment often requires additional procedures, such as diagnostic scans or medication to prevent infection ^50, 63, 69^, blood clots ^47^, transplant rejection ^70^, or epileptic fits ^42^. For example, in the trial by Freed and colleagues, both groups received identical preoperative evaluation, intraoperative sedation and pain control, underwent two PET scans and a MRI scan, and received phenytoin ^42^. In some trials, the same medication was given in both groups, whereas in others unnecessary treatment was omitted or imitated, for example, by injecting saline instead of antibiotics ^71^.

### Standardisation of interventions and care

The active and placebo procedure have to be indistinguishable but they also have to be stable and standardised. Standardisation of the procedure itself may be difficult but is important because surgeons vary in their experience and gain experience throughout the trial.

Some of the changes observed in a trial may not be related to the treatment or the placebo intervention, but may be caused by the natural course of the disease, spontaneous remissions or fluctuations in the severity of symptoms or regression to the mean ^34, 72^. Some changes may be a result of just being in a trial either because of lifestyle changes that are part of the protocol such as self-monitoring, using diaries, or avoiding alcohol, or due to so called “Hawthorne effect”, which refers to change in the behaviour when people, both patients and doctors, know that they are being observed ^34^. Finally, it has been demonstrated that adhering to a protocol improves the performance of doctors, and that patients who adhere to treatment regimens have better outcomes ^73^. Therefore, it is important to standardise pre- and post-operative care, and the explanations given to the patients. For example, in a trial by Sihvonen and colleagues, all procedures were standardised and recorded on video; the post-operative care was also standardised, and all patients received the same exercise program and walking aids ^55^.

### Blinding after the surgery

Most trials blinded the assessors while the surgeon and other staff in the operating room were aware of the group assignment, and did not participate in further treatment, post-operative care or follow-up of the patient ^43, 55, 74^. In a trial by Thomsen and colleagues, the post-operative care and assessment was done at a different hospital than the surgery ^28^. In a trial by Cotton and colleagues, the post-operative care was provided by the referring physician, who was blinded when deciding on treatment, and when this was not sufficient, by the evaluating physician at the study site (who was also blinded) ^74^.

### Bias specific to surgical trials

There are other types of bias that are specific to surgical trials. They are mostly related to patients not being entered into the trial because their symptoms are too severe or not severe enough to justify surgery or because the anatomical conditions or technical difficulties make the surgery impossible to perform. For example, in trials on upper gastrointestinal tract bleeding, the endoscopic procedure was not performed if the rate of blood loss was too fast, or the endoscopy was judged to be life-threatening and posed an unacceptable risk ^75– 77^. This meant that only patients with less severe symptoms were included in the study. In other trials, some patients were excluded because they could not tolerate endoscopy, or due to anatomical conditions that made the surgery impossible to carry out, for example, not being able to aim a laser at the bleeding arteries ^77^. Alternatively, some patients were not included in a trial because they were no longer eligible, for example because the bleeding had stopped ^77^ or they no longer reported the symptoms on the day of the study ^78^. These confounders are difficult to predict and control for.

## Conclusions

Blinding in trials of interventional procedures is possible and many creative methods have been used to maintain the blinding. Interventional procedures are challenging to blind, but the effort is worthwhile because of the obvious benefits, such as avoiding bias, as well as the less evident benefits, such as avoiding patient drop-out in the control arm.
