# Supplementary material for: Blinding in trials of interventional procedures is possible and worthwhile
Source: F1000Res. 2018 Jan 30;6:1663. Originally published 2017 Sep 8. [Version 2] doi: 10.12688/f1000research.12528.2 (PMC5717470; doi:10.12688/f1000research.12528.2)
Supplement: Peer review history

# Peer review: Referee response for version 1

**DOI:** 10.5256/f1000research.13566.r25901

**Published:** 2017-12-04

**Authors:** Asbjørn Hróbjartsson, Helene Moustgaard

**Affiliations:** 1 Centre for Evidence-Based Medicine, Odense University Hospital (OUH), University of Southern Denmark, Odense, Denmark

The overall topic of this paper is important: Blinding facilitates unbiased trial results, but blinding is less used in surgical trials (and less frequently discussed) than in trials of pharmacological interventions. A prevalent preconception is that blinding is not possible, or not relevant, in surgical trials. The main strength of the paper is that it illustrates well this to be ill founded.

 However, we wondered whether the manuscript could be further improved. First, we were in doubt what the specific aim of the paper was. We assumed that the authors aimed to discuss the methodological role of binding in surgical trials (and not primarily to present and reflect on their previous review of surgical placebo trials)? If so, it would have strengthened the paper if it had provided some reflections on the barriers to the use of blinding in surgical trials, the ethical issues involved (and how we can best balance the need for reliable evidence for effects of surgery against the risks or discomforts patients may be exposed to in blinded surgical trials), as wells as discuss what measures could be taken to remedy the current situation where blinding is used infrequently in surgical trials. In general, the reader would be better prepared if the paper clearly stated whether the topic it addresses is blinding in surgical trials or use of placebo in surgical trials and whether the main focus is providing practical examples or providing a general overview of the topic.

 It would be helpful if the paper defined central terms used. For example, what is meant by “interventional procedures”? Does that include acupuncture, radiotherapy, and physiotherapy?  Also, how do the authors conceptualize “subjective outcomes vs. objective outcomes” [1].

 The central section *Bias specific to surgical trials* appears compressed. It would help the reader if the section could more clearly show how the example provided introduces bias, and in what way this bias is specific too surgery (rather than just more frequently encountered).

 Of minor importance was that we were puzzled by some of the references used. For instance, in the *Introduction*, there is a reference to a study by Wood et al [2], but several more recent studies have looked at this issue [3]. Also, in the section *Blinding of outcome assessors *the authors refer to a study by one of the undersigned, to the effect that the study documents a “Pygmalion effect” in un-blinded studies [4]. The term, however, was not used in the original publication. It would possibly help the reader if the authors referred to publications that provide a general overview of blinding in trials (and not only surgical trials).

We have read this submission. We believe that we have an appropriate level of expertise to confirm that it is of an acceptable scientific standard.

## References

[1] MoustgaardHBelloSMillerFGHróbjartssonA: Subjective and objective outcomes in randomized clinical trials: definitions differed in methods publications and were often absent from trial reports. *J Clin Epidemiol*.2014;67(12) : 10.1016/j.jclinepi.2014.06.020 1327-34 10.1016/j.jclinepi.2014.06.020 25263546

[2] WoodLEggerMGluudLLSchulzKFJüniPAltmanDGGluudCMartinRMWoodAJSterneJA: Empirical evidence of bias in treatment effect estimates in controlled trials with different interventions and outcomes: meta-epidemiological study. *BMJ*.2008;336(7644) : 10.1136/bmj.39465.451748.AD 601-5 10.1136/bmj.39465.451748.AD 18316340PMC2267990

[3] PageMJHigginsJPClaytonGSterneJAHróbjartssonASavovićJ: Empirical Evidence of Study Design Biases in Randomized Trials: Systematic Review of Meta-Epidemiological Studies. *PLoS One*.2016;11(7) : 10.1371/journal.pone.0159267 e0159267 10.1371/journal.pone.0159267 27398997PMC4939945

[4] HróbjartssonAThomsenASEmanuelssonFTendalBHildenJBoutronIRavaudPBrorsonS: Observer bias in randomised clinical trials with binary outcomes: systematic review of trials with both blinded and non-blinded outcome assessors. *BMJ*.2012;344:e1119 2237185910.1136/bmj.e1119

# Response

**Published:** 2018-01-14

**Authors:** Karolina Wartolowska

**Affiliations:** University of Oxford, UK

Our aim was to discuss the methodological role of blinding in surgical trials and to demonstrate that blinding can and should be done in surgical trials, especially, if the outcomes are subjective. The need to control for bias in surgical trials is often not recognised. We agree with the Reviewer that there is a preconception/assumption that blinding in surgical trials is not possible or not necessary. Surgery may mean an open surgery undertaken to preserve life. In such cases the outcome is objective, binary and with low risk of bias. However, nowadays, many invasive procedures are performed not to save lives but to improve the quality of life, function or to reduce pain. As a consequence, their outcomes are highly subjective, and as such, prone to bias. This problem seems to be unappreciated in clinical and research community. In this paper, we wanted to demonstrate that blinding can be achieved in surgical trials and that it is necessary if the outcomes are subjective.

Placebo-controlled surgical trials represent a single type of trial that requires blinding, but similar arguments apply to trials comparing two different surgical methods.

In this paper, we did not discuss the placebo because our main focus was blinding as a way to control bias. We used placebo-controlled surgical trials as an example but similar arguments apply to trials comparing two different surgical methods. We have already written about barriers in completing placebo-controlled surgical trials (Wartolowska *et al.*, 2016), about the balance between harms and benefits of such trials (Wartolowska *et al.*, 2014) and about the ethical implications of placebo in surgery (Savulescu, Wartolowska and Carr, 2016)(George *et al.*, 2016).

We have used acupuncture as an example of an experimental study on placebo effect because acupuncture is the only type of invasive procedure used in such studies.

We have included radiofrequency procedures because they change anatomy. In this paper, we have defined surgery as “any interventional procedure that changes the anatomy and requires a skin incision or the use of endoscopic techniques; dental studies were excluded. We used the term placebo to refer to a surgical placebo, a sham surgery, or an imitation procedure intended to mimic the active intervention; including the scenario when a scope was inserted and nothing was done but patients were sedated or under general anaesthesia and could not distinguish whether or not they underwent the actual surgery”.(Wartolowska *et al.*, 2014) We have added these definitions to the manuscript.

“Outcomes were classified as “subjective”, i.e., patient-reported and depending on the patients’ perception and cooperation, “assessed”, i.e., subjective ratings by external assessors and depending upon their judgment, and “objective”, i.e., measured using devices or laboratory tests and independent of patients’ or observers’ perception, for example, weight.”(Wartolowska *et al.*, 2016)

As suggested by the Reviewers, we have explained in the section titled “Bias specific to surgical trials” that by including only patients with non-life-threatening symptoms or with sufficiently severe symptoms on the day of surgery a bias is created, which is difficult to control for.

As suggested by the Reviewers, we have added the paper by Page et al. as a reference in this paragraph. We have also added a reference that provides a general overview of blinding in trials (Hróbjartsson and Boutron, 2011) and provided references to Rosenthal’s papers to explain the “Pygmalion effect”.(Rosenthal and Jacobson, 1968)

**REFERENCES**

George, A. *et al.* (2016) ‘When should placebo surgery as a control in clinical trials be carried out?’, *The Bulletin of the Royal College of Surgeons of England*, 98(2), pp. 75–79.

Hróbjartsson, A. and Boutron, I. (2011) ‘Blinding in randomized clinical trials: Imposed impartiality’, *Clinical Pharmacology and Therapeutics*. Nature Publishing Group, 90(5), pp. 732–736.

Rosenthal, R. and Jacobson, L. (1968) ‘Pygmalion in the classroom’, *The Urban Review*, 3(1), pp. 16–20.

Savulescu, J., Wartolowska, K. A. and Carr, A. (2016) ‘Randomised Placebo-Controlled Trials of Surgery: Ethical Analysis and Guidelines’, *J Med Ethics*, 42(12), pp. 776–783.

Wartolowska, K. *et al.* (2014) ‘Use of placebo controls in the evaluation of surgery: systematic review.’, *BMJ (Clinical research ed.)*, 348(May), p. g3253.

Wartolowska, K. *et al.* (2016) ‘Feasibility of surgical randomised controlled trials with a placebo arm: a systematic review’, *BMJ Open*, 6(3), p. e010194.

Wartolowska, K. A. *et al.* (2016) ‘The Magnitude and Temporal Changes of Response in the Placebo Arm of Surgical Randomized Controlled Trials - A Systematic Review and Meta-Analysis’, *Trials*. Trials, 17(1), p. 589.

# Peer review: Referee response for version 1

**DOI:** 10.5256/f1000research.13566.r28006

**Published:** 2017-12-04

**Authors:** Harald Walach

**Affiliations:** 1 Department of Pediatric Gastroenterology, Medical University Poznan, Poznań, Poland

This review is, as its self-set goal goes, very well substantiated, balanced and timely. There is no factual correction in itself that is necessary in my view.

 However, I wish to give a few critical feedbacks to the authors that they may wish to integrate. I would prefer if they did, but in my view it is unfair to formally demand such an extension. However, the debate itself would profit.

 My point is regarding the *presuppositions* of the current model the authors implicitly subscribe to, for instance when they say randomised trials “provide the highest level of evidence” (1 ^st^ para). This is true, but only from a  - debated – point of departure that accepts the hierarchisation of evidence as a given and true proposal. As it happens, we, and others, have debated this [1,2]. The hierarchisation of evidence accepts – without further reflection – that internal and external validity are compatible, linear concepts, where first internal evidence can be gleaned and is prior and superior to external validity. This I find an unsubstantiated point of view that needs at least acknowledgement. All the arguments in this paper address the improvement of internal validity, and as such they are very good.

 The argument that “specific” effects have to be tested against “non-specific” or placebo-effects and therefore blinding is necessary also makes presuppositions that are not clarified and critically debated. The presupposition is that there is such a thing as separability of these effects. This separability argument is highly speculative, very little researched and where and when researched found to be wrong [3, 4]. One can, of course, try and disentangle various components of interventions. And especially where interventions are costly or fraught with side-effects this is even an ethical requirement, because else patients would be submitted to problematic procedures with little benefit or where other interventions might be just as good, all taken together. But one should be clear about the fact that this is not something that automatically gets rid of all problems in evaluating therapeutic procedures.

 Therefore, I think the arguments the authors present are valid within the context of improving internal validity of intervention trials that offer costly and potentially dangerous or problematic interventions. But I think the authors should be clear themselves that this is not a universally valid strategy and it would be wise to integrate some caveats and restrictions in their argument.

I have read this submission. I believe that I have an appropriate level of expertise to confirm that it is of an acceptable scientific standard.

## References

[1] WalachHLoefM: Using a matrix-analytical approach to synthesizing evidence solved incompatibility problem in the hierarchy of evidence. *J Clin Epidemiol*.2015;68(11) : 10.1016/j.jclinepi.2015.03.027 1251-60 10.1016/j.jclinepi.2015.03.027 26148834

[2] GreenhalghTHowickJMaskreyNEvidence Based Medicine Renaissance Group: Evidence based medicine: a movement in crisis?. *BMJ*.2014;348:g3725 2492776310.1136/bmj.g3725PMC4056639

[3] WalachH: Placebo controls: historical, methodological and general aspects. *Philos Trans R Soc Lond B Biol Sci*.2011;366(1572) : 10.1098/rstb.2010.0401 1870-8 10.1098/rstb.2010.0401 21576144PMC3130405

[4] MeissnerKFässlerMRückerGKleijnenJHróbjartssonASchneiderAAntesGLindeK: Differential effectiveness of placebo treatments: a systematic review of migraine prophylaxis. *JAMA Intern Med*.2013;173(21) : 10.1001/jamainternmed.2013.10391 1941-51 10.1001/jamainternmed.2013.10391 24126676

# Response

**Published:** 2018-01-14

**Authors:** Karolina Wartolowska

**Affiliations:** University of Oxford, UK

We are grateful for the comment regarding the hierarchy of evidence. We have rephrased the sentence so that it now reads “high level of evidence because they attempt to minimise bias due to confounding factors”. We have also added the suggested reference (Walach and Loef, 2015).

We agree that RCTs have high internal validity because they are performed in a standardised way, and because they attempt to control for confounding factors. However, owing to the controlled conditions under which they are performed, RCTs often have low external validity. In this paper, we wanted to draw attention to the fact that surgical trials often use subjective outcomes, and are therefore prone to bias, which has to be minimised or controlled for.

We agree with the Reviewer, that the “specific” and “non-specific” effects are difficult to separate or to unequivocally define. As surgical procedures are complex and consist of many elements, it is sometimes difficult to identify the crucial surgical element that is believed to be therapeutic. We have described the CSAW trial as an example. The aim of this trial was to investigate whether the improvement was due to change of anatomy (arthroscopic removal of a bony spur on the acromion) or it was not related to the change in anatomy and the spur removal was not necessary because a placebo procedure (arthroscopy only) results in similar change in pain and function.

We wanted to draw attention to the fact that in the case of surgical trials, with their high costs and risks, one would expect to see a much larger effect after a surgical procedure than after a placebo or no treatment.

We are familiar with the debate in the literature regarding specific and non-specific effects. In the context of surgical trials, by “specific effects” we meant changes directly related to change in anatomy, for example, a bony spur removal in the trial of arthroscopic shoulder decompression surgery for subacromial pain. All other possible factors resulting in improvement, such as the effect of arthroscopy, the placebo effect, influence of patient-doctor interactions, symptoms fluctuation as well as spontaneous improvement were regarded as “non-specific”. We also agree with Enck and colleagues that the effect in the placebo and active arms may not be additive; therefore, the improvement related to the crucial surgical element may not equal the difference between the effect in the active and placebo arms.(Enck *et al.*, 2011) We would like to clarify that blinding and placebo control help to minimise bias and aid in the interpretation of trial results; especially if the outcomes are subjective and the symptoms fluctuate or may spontaneously improve.

**REFERENCES**

Enck, P. *et al.* (2011) ‘The placebo response in clinical trials: more questions than answers.’, *Philosophical transactions of the Royal Society of London. Series B, Biological sciences*, 366, pp. 1889–1895.

Walach, H. and Loef, M. (2015) ‘Using a matrix-analytical approach to synthesizing evidence solved incompatibility problem in the hierarchy of evidence’, *Journal of Clinical Epidemiology*. Elsevier Inc, 68(11), pp. 1251–1260.

# Peer review: Referee response for version 1

**DOI:** 10.5256/f1000research.13566.r25898

**Published:** 2017-09-20

**Authors:** Ian A Harris

**Affiliations:** 1 Whitlam Orthopaedic Research Centre, Ingham Institute for Applied Medical Research, South Western Sydney Clinical School, University of New South Wales, Liverpool, NSW, Australia

This manuscript is a narrative overview of methods of blinding in trials of procedures, using the previous review by these authors as the source of data. It explains the rationale for blinding and gives examples (subgrouped) of how blinding was achieved in previous trials. The authors wisely avoid a detailed discussion of the placebo effect and concentrate on the need for blinding based on empirical evidence of bias resulting from not blinding.

 The paper reinforces the need for blinding in procedural trials which is a reasonable demand that is increasingly being heeded. In other words, the message is important.

 The paper is very well written. I only have minor comments, below:

1. Page 2, second last paragraph, “crucial element of believed to be the cure” should read  “crucial element believed to be the cure”
2. The evidence of bias resulting from not blinding may be one sided. Opposing evidence should be discussed, such as Berkmann ND, The Empirical Evidence of Bias in Trials Measuring Treatment Differences ^1^ which shows that the effect of blinding may even be in the opposite direction and the classic Schultz article from 1995 ^1^ which gives a lower estimate of effect exaggeration from unblinding. There are several other summaries and meta-epidemiological studies in this area.

I have read this submission. I believe that I have an appropriate level of expertise to confirm that it is of an acceptable scientific standard.

## References

[1] BerkmanNDSantaguidaPLViswanathanMMortonSC: The Empirical Evidence of Bias in Trials Measuring Treatment Differences. 25392898

[2] SchulzKFChalmersIHayesRJAltmanDG: Empirical evidence of bias. Dimensions of methodological quality associated with estimates of treatment effects in controlled trials. *JAMA*.1995;273(5) :408-12 782338710.1001/jama.273.5.408

# Response

**Published:** 2018-01-14

**Authors:** Karolina Wartolowska

**Affiliations:** University of Oxford, UK

We are grateful for the comment and we have added an explanation that trials have limited external validity, as by controlling for confounding factors and standardising the trial conditions, they become very different from usual clinical practice. They are also liable to have study populations that are so unique that the results of the trial cannot be easily generalised.

In the case of blinding, the magnitude of effect may be lower in a blinded trial than in everyday practice, because blinding causes uncertainty about treatment allocation. In clinical practice, there is usually no doubt as the patient-doctor relationship is based on trust and on the assumption that doctors offer patients the best available treatment. In an RCT, there is an inherent uncertainty as to which treatment group a patient has been allocated.(Enck *et al.*, 2011)

**REFERENCES**

Enck, P. *et al.* (2011) ‘The placebo response in clinical trials: more questions than answers.’, *Philosophical transactions of the Royal Society of London. Series B, Biological sciences*, 366, pp. 1889–1895.
